# The dilemmas and countermeasures of postgraduate management from the perspective of oriental management

**DOI:** 10.3389/fpsyg.2025.1749637

**Published:** 2026-01-21

**Authors:** Jun Hu

**Affiliations:** Office of the Headmaster, Jiangsu University of Technology, Changzhou, China

**Keywords:** oriental management, graduate students, humanistic care, psychological pressure, five behaviors

## Abstract

Postgraduate education provides technological and talent support for the growth of China’s high-quality economic. Scientific and rational management is an important way to ensure the quality of postgraduate education. There are some dilemmas by traditional hierarchical management model for postgraduates. This study investigates the countermeasures from the perspective of Oriental Management. It can innovate the postgraduate management philosophy by applying the “Five Behaviors” of Oriental Management (i.e., humanistic behavior, psychology behavior, relationship behavior, strategy behavior and human resource behavior).

## 1 Introduction

Postgraduate education is an important way for China to accelerate the implementation of the innovation-driven development strategy and cultivate high-level, top-class innovative talents (Jiang and Wang, 2025). With the popularization of higher education in our country, graduate students are gradually expanding their enrollment (Chen, 2004). The expansion of enrollment has made the structure of postgraduates more diverse, with individual differences and various demands. Postgraduate management plays an important role in helping them complete their studies successfully.

Humanistic care can stimulate students’ creativity in learning and promote their comprehensive development (Yang, 2018). In postgraduate management, it is necessary to strengthen the humanistic care. Raising the humanistic care to the theoretical level of management can be explained by Oriental management. Oriental management is a discipline that promotes the coordinated development of individuals or organizations based on the integration and innovation of management ideas from ancient to modern times, both domestically and internationally (Su and Duan, 2019). Oriental Management was innovatively and systematically transformed by Mr. Su Dongshui, Chief Professor of Fudan University, who integrated the Chinese ideological and cultural heritage that has been accumulated for several 1,000 years into management theory. Based on the theoretical and practical foundations of Chinese management, Western management and Chinese business management. He summarized Oriental Management theory with the core ideas of “Three For”: putting people in the first place, regarding morality as priority, and behaving oneself so as to serve others (Xiong and Yu, 2011). The “Five Behaviors” of Oriental Management refer to the five methods that operate in the leadership process: humanistic behavior, psychology behavior, relationship behavior, strategy behavior and human resource behavior. These Five Behaviors are specific manifestations of Oriental Management in the practical stage (Wu, 2009). It becomes an important component of modern Chinese management theory. At present, there are few studies about integrating the “Five Behaviors” into postgraduate management.

This paper selects J University in a prefecture-level city in Jiangsu Province, a major educational province in China, which has over 10 years of postgraduate education experience, as an empirical case. J University has postgraduate students from abroad and has extensive cooperation and exchanges with universities in the Belt and Road countries. In order to understand the psychological pressure and humanistic care of postgraduates, a questionnaire survey was conducted among postgraduates of J University. The set questions were all non quantitative form topics, which were sent to postgraduates in the form of a Sojump electronic questionnaire. A questionnaire titled “Postgraduate Education and Management” was designed and conducted a questionnaire survey. A total of 116 questionnaires were distributed and 110 valid questionnaires were collected, with an efficiency rate of 94.82%. Since the questionnaires are all non quantitative forms, reliability and structural validity analysis cannot be conducted.

## 2 Discussion

### 2.1 The dilemmas in postgraduate management

#### 2.1.1 Absence of a people-oriented management philosophy

The management of student focuses more on the rigid constraints, lacking humanistic care. It mainly manifested in the following two aspects. Firstly, managers have not fully considered the personalized development needs of postgraduate. They are usually between the ages from 22 to 35, with a wide range of sources and have significant differences in their life experiences and family backgrounds. Some of them even have work experiences. Managers emphasize their own authority, and execute work tasks in a commanding manner. They adopt a “one size fits all” approach in management, focusing on constraints for graduate students. The personalized development needs of graduate students are difficult to meet.

Secondly, the service awareness is correspondingly weak. Graduate students are not putted in the first place. While mangers simply obeying the work arrangements of superiors and aiming to satisfy the superiors as the work goal, without paying enough attention to the needs of graduate students. There is even a “bureaucratic” mentality among mangers. A postgraduate satisfaction survey showed that management services scored the lowest in satisfaction evaluations among course teaching, scientific research training, guidance teachers, and management services. Graduate students have relatively low satisfaction with dormitories and employment guidance, with satisfaction rates of 55.0 and 54.97%, respectively (Chen, 2024). Because with the expansion of postgraduate enrollment, some universities have limited dormitory resources, and a few universities do not provide dormitories for some graduate students.

This study conducted a questionnaire survey on graduate students at J University. 84.55% of the respondents chose “Yes” to the question “Do you think it is necessary to enhance teachers’ humanistic care for students,” while only 15.45% chose “No.” This indicates that the majority of postgraduates believe that humanistic care is insufficient and there is an urgent need to enhance it.

#### 2.1.2 Psychological health problems

The learning experiences, life experiences and social relationships of postgraduates are more complex than those of undergraduates, and they are given higher expectations by their families, schools and society. The continuous expansion of enrollment has made the student source structures, psychological conditions and personalized development needs of postgraduates more diverse, and the employment pressure has also increased. It brings many new challenges to postgraduate management. The proportion of postgraduates suffering from depression or anxiety is significantly higher than the general population (Evans et al., 2018). According to the survey results of “Postgraduate Psychological Health Status and Influencing Factors in 2019,” among the surveyed postgraduates, 35.5% may have some degree of depressive symptoms and 60.1% have anxiety problems (JYB, 2021). The psychological health problems of postgraduates are severe. As shown in Supplementary Table 1, the questionnaire survey conducted among postgraduates of J University found that 87.27% of postgraduates experienced varying degrees of psychological pressure during their postgraduate studies, among which 46.36% were in a state of mild psychological pressure, and only 12.73% had no psychological pressure at all. It means that most postgraduates experience varying degrees of psychological pressure.

For graduate students’ psychological health problems, managers tend to focus more on afterwards remedial measures when the problems have already manifested, such as psychological intervention and counseling. This management approach ignores prior prevention and cannot fundamentally reverse the severe situation of students’ psychological problems. It is also difficult to form a long-term student psychological health management model.

#### 2.1.3 Lack of equal and effective communication

Postgraduate management is a process of interaction between teachers and students, requiring communication between them. Effective communication is the best way to build relationships. Research has shown significant relationships between high-quality student-teacher interactions and students’ self-efficacy, emotional regulation and graduate aspirations, but inadequate relationships and interactions negatively influence these domains (Jiang et al., 2025). Due to the deep-rooted regulatory mindset, the relationship between teachers and students is usually presented in the form of superiors and subordinates. Superior are accustomed to giving orders, and graduate students usually passively listen to their requests and suggestions. Over time, superiors may not fully understand the thoughts and perspectives of the graduate students.

If the manager is in a high position and the student is in a low position during communication, it becomes a barrier to effective communication. Especially for postgraduate mentors, they are the “first responsible person” for the cultivation of postgraduates and usually have profound attainments in a certain academic field. Mentors are accordingly endowed with a relatively higher social status, playing a crucial role in the growth and development of postgraduates. When communicating with postgraduates, mentors tend to show a sense of “authority,” causing postgraduates to be reluctant to fully express their thoughts and demands. Postgraduates may develop a resistant mentality and be unwilling to communicate with their mentors gradually. Some mentors spend less time in guiding postgraduates’ experiments than “running for funds” and “running for projects” (HBSKW, 2020). Through interviews with several class monitors of postgraduates of J University, it was found that the phenomenon of postgraduates applying to change mentors not unusual. Some graduate students have tense teacher-student relationships due to poor communication with their mentors. However, postgraduates have a strong sense of self-esteem, equality and self-actualization as high-level talents.

#### 2.1.4 Deficiencies in management strategies

Management is both a science and an art, emphasizing methods and strategies. Managers neglect to draw strategic wisdom from excellent traditional Chinese culture. They are accustomed to using past experience, with the aim of completing work tasks assigned by superiors. They carry out their works by issuing administrative instructions, requiring students to obey the regulations, which restricts the personalized development of graduate students.

Humble acceptance is the foundation of successful decision-making, but under a hierarchical management system, managers rarely pay attention to the feedback and guidance of graduate students, ignoring the main position of postgraduates, and cannot think from graduate student’s perspective. For example, in the process of policy formulation, managers failed to give full play to students’ subjective initiative, did not invite postgraduate representatives to participate in policy formulation, and did not fully listen to the reasonable suggestions from postgraduates. The policies formulated have deviated from the actual situation of graduate students, resulting in low implementation efficiency and failure to achieve the expected results.

#### 2.1.5 Grassroots managers lack of work enthusiasm

With the increasing scale of graduate students, the workload of grassroots managers has greatly increased, new situations and problems emerge one after another. However, the salary and benefits of grassroots managers have not increased accordingly. The management of Chinese graduate students usually adopts a joint management model of university graduate schools and secondary colleges. Mainly managed by secondary colleges, supplemented by graduate schools. Compared with the undergraduate management team, the postgraduate management team is not as well-established. According to the “Regulations on the Construction of the Counselor Team in Regular Institutions of Higher Learning” issued by the Ministry of Education of China, “Universities should be equipped with full-time counselors at a ratio of no less than 1:200 of the student population (MOE, 2017).” But many secondary colleges do not meet the standard for equipping full-time counselors due to their graduate student scale, and usually only have one postgraduate secretary who is responsible for a series of complex tasks such as postgraduate recruitment, academic affairs, employment, and daily management, from enrollment to graduation.

The setting of administrative levels in university management positions presents a “pyramid” shape, and the career development of grassroots managers mainly depends on the promotion of administrative levels. Universities often attach great importance to the teaching and research work for graduate students, but pay insufficient attention to the construction of management teams. Grassroots managers have a much lower social status than full-time teachers and researchers. It is difficult for grassroots managers to make breakthrough progress in their work, and there are relatively few opportunities for job promotion. In addition, due to numerous affairs, there is not enough time to write and publish academic papers in educational management journals or apply for research projects. Thus, the grassroots managers are difficult to meet the conditions for promotion to senior professional titles. There are also fewer opportunities for grassroots graduate managers to participate in further education or training to improve their professional skills and qualities. Research has shown that only 30.1% of graduate management personnel believe that their work enthusiasm has “continuously improved” since engaging in this job, 58.1% have “remained basically unchanged,” and 11.8% have “gradually decreased” (Pang et al., 2008).

### 2.2 Countermeasures from the perspective of “five behaviors” in oriental management

#### 2.2.1 Humanistic behavior: putting people in the first place

The humanistic behavior in Oriental Management means to respect the ultimate goals and personal values of individuals, follow the principles and laws of human development, it regard the development, improvement, and self realization of individuals as the highest value (Yu and Su, 2010). The humanistic behavior emphasizes putting people in the first place. It is one of the core ideas of Oriental Management. Putting people in the first place is a shift in value orientation, it aims at achieving the comprehensive development of individuals. The greatness of a university lies in its students, who are the fundamental reason for a university’s existence and the main subjects of its educational and teaching activities. Managers should respect the factors influencing individual graduate students’ innovative and creative abilities—such as interest, curiosity, and imagination—understand their personality differences, and meet their diverse needs. For example, addressing the needs of graduate students in areas such as accommodation and career guidance. Graduate students can devote more time and effort to their studies after their needs are met.

In the management process, it is necessary to follow the laws of graduate growth and development, as well as the laws of scientific research activities. Managers who want graduate students to develop in the expected direction should not blindly criticize or mechanically constrain them. Managers should change the traditional hierarchical management model and shift from efficiency oriented instrumental rationality to people-oriented value rationality. It should be noted that putting people in the first place does not mean ignoring various rules and regulations. When graduate students engage in illegal behavior, they should still be criticized and educated in time, while paying attention to the methods to avoid exacerbating conflicts.

#### 2.2.2 Psychology behavior: providing personalized psychological services

The psychology behavior in Oriental Management refers to management psychology, addressing the complex psychological contradictions of people, and cultivating a healthy psychological state. Managers care for postgraduates’ pressure and difficulties initiatively, such as learning, research and employment. When graduate students experience emotions such as fear, anxiety, and tension, managers should promptly provide personalized emotional comfort and psychological counseling. Managers buffer these stress among postgraduates through emotional support, academic tolerance and resource protection (Hu et al., 2025). Managers also need to communicate with the school’s psychology health center, which should identify and judge the psychological problems of postgraduates, establish a “one person, one file” psychological health record, and carry out personalized psychological counseling. Through the psychological consultation hotline, psychological health lectures, and WeChat official account to achieve full coverage of postgraduate psychological health education. Managers and psychology health centers work together to create a positive, optimistic, and upward psychology health campus environment, cultivating the spiritual qualities of postgraduates to resolve psychological problems and accept themselves.

In addition to promoting psychology health education and providing psychological counseling, psychology health center should also dedicated to research on the psychology characteristics of postgraduates by conducting sample analysis, grasp the patterns of psychology problems, prioritize intervention in psychology health, and evaluate the effectiveness of psychological intervention methods. Thus, the quality and effectiveness of graduate students’ mental health work can be improved.

#### 2.2.3 Relationship behavior: building a positive and interactive relationship

Oriental Management focuses on the interpersonal relationships reflected in communication, emphasizing harmony, trustworthiness and honesty. Mentors’ guidance for postgraduates involves various aspects such as academic research, personal life and career development. It is necessary to establish a good relationship between mentors and postgraduates. For example, favorable mentor-postgraduate interactions and relationships provide important psychosocial buffers against psychological disorders (e.g., anxiety, burnout) and negative emotions for medical postgraduates (Jiang et al., 2025). Research has shown that the way of mentor-postgraduate interaction has a significant impact on the knowledge acquisition effect of postgraduates and the “good teachers and helpful friends.” This mentor-student interaction method has the most ideal effect on knowledge acquisition (Xie et al., 2020). Mentors should respect the innovative consciousness of postgraduates and encourage them to propose new ideas. The mentoring relationship accompanies the entire postgraduate learning stage. Mentors demonstrate friendliness and tolerance during guidance, listening to students’ viewpoints with an open and supportive attitude. This inclusive mentor style can better promote the occurrence of innovative behavior among postgraduates (Wang et al., 2021). Mentors demonstrate an inclusive mentor style when providing research guidance, academic assistance, and employment guidance. Maintain harmonious and friendly interactions between mentors and graduate students to prevent the occurrence of subordinate, employment, and even hostile relationships.

Universities should not only focus on the academic achievements of mentors, but also on their graduate management abilities. A more comprehensive evaluation framework should be established by reforming the practice of evaluating mentors solely based on indicators such as research projects and received funds. This framework should pay more attention to the guidance time, guidance frequency, on-time graduation rate, employment success rate, etc. for graduate students, and encourage mentors to focus their main energy on guiding graduate students. Mentors provide assistance for graduate students’ career and professional development, offering emotional and psychological support (Wang and Liu, 2023). By creating incentive mechanisms that encourage mentors to dedicate more time and effort to student guidance, universities can improve the overall quality of postgraduate education and ensure that students receive the necessary support to succeed both academically and professionally. Furthermore, postgraduates need to communicate with their mentors and fellow apprentices actively, seek their academic guidance, and enhance the emotional connection within the research group.

#### 2.2.4 Strategy behavior: drawing managerial wisdom from excellent traditional Chinese culture

Strategy behavior is a unique managerial approach in Oriental Management, which refers to the comprehensive application of methods and techniques by managers to achieve expected goals. Strategy behavior contains profound philosophical ideas. It is closely related to 1,000 years of excellent traditional Chinese culture. Managers should draw on the managerial wisdom contained in the culture.

The principle of “valuing righteousness over profit” is the essence of excellent traditional Chinese culture. It requires managers to guide postgraduates to correctly treat morality and interests, and cultivate the noble character that value righteousness over profit. The “Doctrine of the Mean” is the standard and norm of Chinese managerial wisdom, emphasizing impartiality. Managers should maintain balance when dealing with problems, neither being extreme nor conservative, achieving the goal of harmony and stability. The thought of “outer circle and inner square” advocates management combining rigidity with flexibility (Zhou, 2017).

“By drawing on the wisdom of the masses, nothing is impossible” inspires managers to brainstorm and allow postgraduates to participate in the policy-making process, valuing the wisdom of the postgraduate community. For example, when formulating the methods and implementation rules for postgraduates awards, managers should pay attention to the rights and interests of stakeholders and invite postgraduate representatives to participate in decision-making and express their opinions. Managers who adopt reasonable opinions proposed by postgraduates can make the formulated system more comprehensive and operable. At the same time, managers need to pay attention to the feedback of postgraduates, shifting from “one-way management” to “two-way governance.”

#### 2.2.5 Talent behavior: selecting and cultivating excellent grassroots managers

Optimize the selection, training and guarantee mechanism for grassroots managers. Firstly, universities need to strictly control the entrance of employment. Postgraduate management has gradually evolved into a specialized job. It requires teachers who possess professional knowledge in administrative management theory and practice, higher education, psychology, etc., familiar with the development laws of postgraduate growth processes, to serve as mentors, postgraduate secretaries, class teachers, etc., creating a good organizational, public opinion and humanistic environment for postgraduate education. Secondly, universities need to attach importance to the cultivation of grassroots managers. For example, university leaders regularly hold symposiums with mentors, graduate student secretaries, class teachers etc., listen to their demands, solve the difficulties and problems at school level. It can reflect that the university leaders’ concern for grassroots managers. University graduate schools and secondary colleges need to create training opportunities for grassroots managers to participate in comprehensive management ability improvement, learn management professional theoretical knowledge. Thirdly, universities ensure the various welfare benefits for grassroots managers corresponding to their workload, performance and contributions, attract and retain more outstanding talents to postgraduate management positions.

## 3 Conclusion

This study innovatively integrates the “Five Behaviors” of Oriental Management, which has great practical guidance value, into postgraduate management. It is conducive to improving the quality of postgraduate education, making postgraduate management wise, warm and rich in connotation.

## Data Availability

The original contributions presented in the study are included in the article/Supplementary material, further inquiries can be directed to the corresponding author/s.
