# Supplementary material for: The dilemmas and countermeasures of postgraduate management from the perspective of oriental management
Source: Front Psychol. 2026 Jan 21;16:1749637. doi: 10.3389/fpsyg.2025.1749637 (PMC12868178; doi:10.3389/fpsyg.2025.1749637)
Supplement: Supplementary file 1 [file Table_1.DOC]

**Appendix**

Tab. 1 Survey results of psychological stress among J university postgraduates

| **Degrees of psychological pressure** | **Proportion** |
| --- | --- |
| destructive psychological stress | 5.45% |
| severe psychological stress | 0.91% |
| moderate psychological stress | 34.55% |
| mild psychological pressure | 46.36% |
| no psychological pressure | 12.73% |
